# Direct lateral maneuvers in hawkmoths

**DOI:** 10.1242/bio.012922

**Published:** 2016-01-06

**Authors:** Jeremy S. M. Greeter, Tyson L. Hedrick

**Affiliations:** Department of Biology, University of North Carolina at Chapel Hill, Chapel Hill, NC 27599, USA

**Keywords:** Free flight, Maneuver, Flight control, *Manduca sexta*, Lateral maneuvers, Sideslip, Side-slip, Dodge, Banked turning, Roll, Flapping counter-torque

## Abstract

We used videography to investigate direct lateral maneuvers, i.e. ‘sideslips’, of the hawkmoth *Manduca sexta*. *M. sexta* sideslip by rolling their entire body and wings to reorient their net force vector. During sideslip they increase net aerodynamic force by flapping with greater amplitude, (in both wing elevation and sweep), allowing them to continue to support body weight while rolled. To execute the roll maneuver we observed in sideslips, they use an asymmetric wing stroke; increasing the pitch of the roll-contralateral wing pair, while decreasing that of the roll-ipsilateral pair. They also increase the wing sweep amplitude of, and decrease the elevation amplitude of, the contralateral wing pair relative to the ipsilateral pair. The roll maneuver unfolds in a stairstep manner, with orientation changing more during downstroke than upstroke. This is due to smaller upstroke wing pitch angle asymmetries as well as increased upstroke flapping counter-torque from left-right differences in global reference frame wing velocity about the moth's roll axis. Rolls are also opposed by stabilizing aerodynamic moments from lateral motion, such that rightward roll velocity will be opposed by rightward motion. Computational modeling using blade-element approaches confirm the plausibility of a causal linkage between the previously mentioned wing kinematics and roll/sideslip. Model results also predict high degrees of axial and lateral damping. On the time scale of whole and half wing strokes, left-right wing pair asymmetries directly relate to the first, but not second, derivative of roll. Collectively, these results strongly support a roll-based sideslip with a high degree of roll damping in *M. sexta*.

## Abstract (precis)

**Summary:** We show that hawkmoths fly sideways by rolling in the direction of movement, adding a left- or right-ward component to their net lift vector. The underlying roll maneuvers are produced from a suite of asymmetric wing kinematic changes and are heavily damped.

## INTRODUCTION

Flying animals must maneuver and stabilize to navigate obstacles and avoid predators when seeking resources and mates. Moths of the *Sphingidae* family provide a ready model system for investigating animal flight maneuverability and stability due to their aerial agility, ease of care/training, large body size, cosmopolitan distribution, and economic significance as agricultural pests. Member species oscillate horizontally while hover-feeding, and rapidly maneuver away if visually startled; such flight behavior may have evolved to avoid ambush predators at flowers (Wasserthal, 1993; Cheng et al., 2011). Previous studies have probed a multitude of sphingid characteristics, including escape flight maneuvers, in detail; but not direct lateral maneuvers, or ‘sideslips’. Here we investigate sideslip kinematics in the sphingid *Manduca sexta* (*L*).

Our first hypothesis was that *M. sexta* sideslip, at least partially, by creating direct lateral force via asynchrony in wing pitch angle, as described in fruit flies (Ristroph et al., 2009). Ristroph et al. (2009) first observed that sideslipping *Drosophila melanogaster* display left-right phase asymmetry in wing pitch rotation. They showed a difference in the wing pitch angle near the end of *Drosophila*'s ≈155° halfstrokes (stroke reversal), where wing trajectories are almost lateral, may create net lateral force that accounts for about half of sideslip acceleration. *M. sexta* wings have similarly quasi-lateral trajectories near the end of their ≈100° halfstrokes, where forces are also high (Bomphrey et al., 2005).

Our alternate hypothesis was that *M. sexta* sideslip solely by rolling to reorient their net aerodynamic force vector. Apparently roll-based, roughly lateral maneuvers during insect hover have been observed, (Ellington, 1984a; Ristroph et al., 2009; Muijres et al., 2015); and recent work on *Aedes aegypti* showed mosquitoes perform direct lateral maneuvers via a simple roll-based rotation of their stroke plane (S. Iams, PhD thesis, Cornell University, 2012). However, *A. aegypti*'s particularly low flapping amplitude of ≈45° may not permit sideslip via wing pitch asynchrony as described in the first hypothesis, since their wings do not achieve roughly opposing lateral trajectories near the ends of strokes like those of fruit flies and hawkmoths.

In this study we found strong agreement between videographic analysis of *M. sexta* lateral maneuvers and first-principles models of our second, roll-only sideslip hypothesis. In further support of this result, we also identified changes to wing kinematics that more fully explain the observed lateral and vertical accelerations. We calculated passive damping time constants for lateral and vertical acceleration based on our fitted equations, and compared them to time constants estimated from computational models.

Further exploring sideslip maneuvers, we next identified wing kinematics associated with roll, added possible passive sources of damping to this model, then tested it against the first and second time derivatives of observed roll orientation. These predictions came from qualitative observations of roll maneuvers, general principles of flapping flight, or previous animal flight maneuvering studies. For all models, we used the corrected Akaike information criterion (AICc), to select the best sets of predictor variables, and *P*-values from our full-parameter test model to confirm coefficient significance.

## RESULTS

### Overview

Our results show that moths use roll to redirect their net force vector and thus initiate lateral maneuvers. They use sweep and elevation amplitude to amplify the force they create, and a mixture of various wing asymmetries to initiate roll. Linear movement is resisted by passive drag, and roll itself is highly damped.

Sideslips were roll-based and largely unidirectional. The average sideslip maneuver, as defined in Materials and Methods, lasted ≈0.2 s. The overall average magnitudes for the first derivatives of yaw, pitch, and roll can be seen in Table S1. During many sideslips, moths experienced brief yaw (and sometimes pitch) rotations, which they later corrected with rotational acceleration in the opposite direction. This explains the relatively high average absolute value for yaw and pitch velocity despite the ideally unidirectional nature of whole-body sideslip maneuvers. Models for vertical and lateral acceleration (*z̈* and *ÿ*) show a relationship consistent with a roll-based lateral acceleration hypothesis. Increases to *ż* and 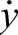 reduce collinear acceleration (*z̈* and *ÿ*). Bilateral increases in sweep and elevation amplitude (*Φ_p_* and *θ_p_*)*_ _* increase vertical and horizontal force production and thus acceleration. These angles, *Φ_p_* and *θ_p_*, are the peak-to-peak angular amplitudes of the wing paths, measured respectively in the horizontal and vertical body reference planes (BRF) for each halfstroke.

**Fig. 1.**
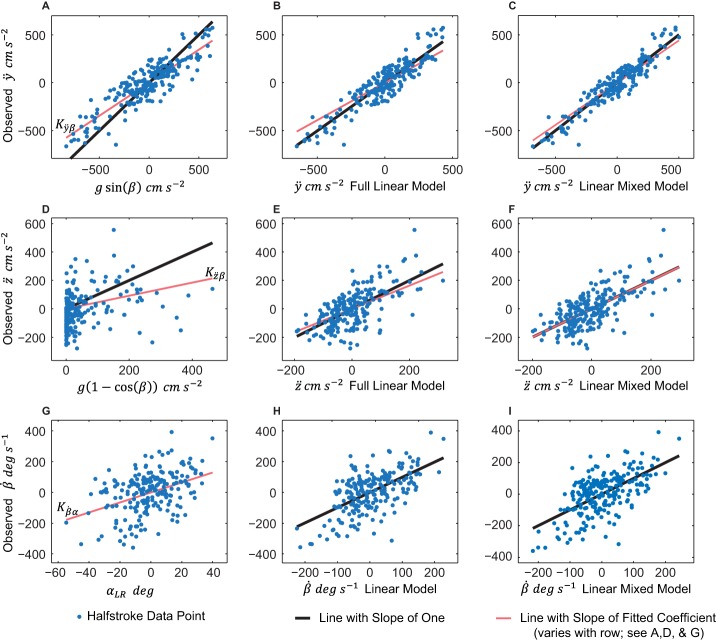
**Three major moth body position and orientation derivative models.** In descending vertical order, rows 1-3 show data for *ÿ*, *z̈*, and 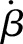. The thin red line has an intercept of zero and a slope equal to the value of the fitted coefficient, (*K_ÿβ_*, *K_z̈β_*, and 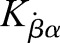 respectively by row). The thicker black line has an intercept of zero and a slope of one. In the first column (A,D,G), we fit *ÿ* and *z̈* to the *a priori* constant dorsally-directed force production model, and 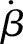 to *α_LR_* (the wing asymmetry that contributed the most to roll velocity). In the second column (B,E,H), we fit *ÿ*, *z̈*, and 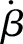 to the complete linear models which resulted from the variable selection process (Eqns 6-8). (C,F) We fit *ÿ* and *z̈* to the full linear mixed models; they differ from column two only by the addition of a random intercept for each moth (which resulted in lower AICc values than the models without this adjustment). (H) Linear model for 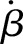, which includes *α_LR_*, *Φ_LR_*, and 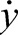. (I) Full linear mixed model for 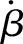 which differs from panel H by the addition of separate up- and downstroke coefficient estimates for elevation angle. It is important to note that, while we do present this data, panel H scored better than panel I in AICc analysis. *n*=218 halfstrokes from 19 maneuvers from 4 moths. For *P*-values see Tables 1 and 3.

**Fig. 2.**
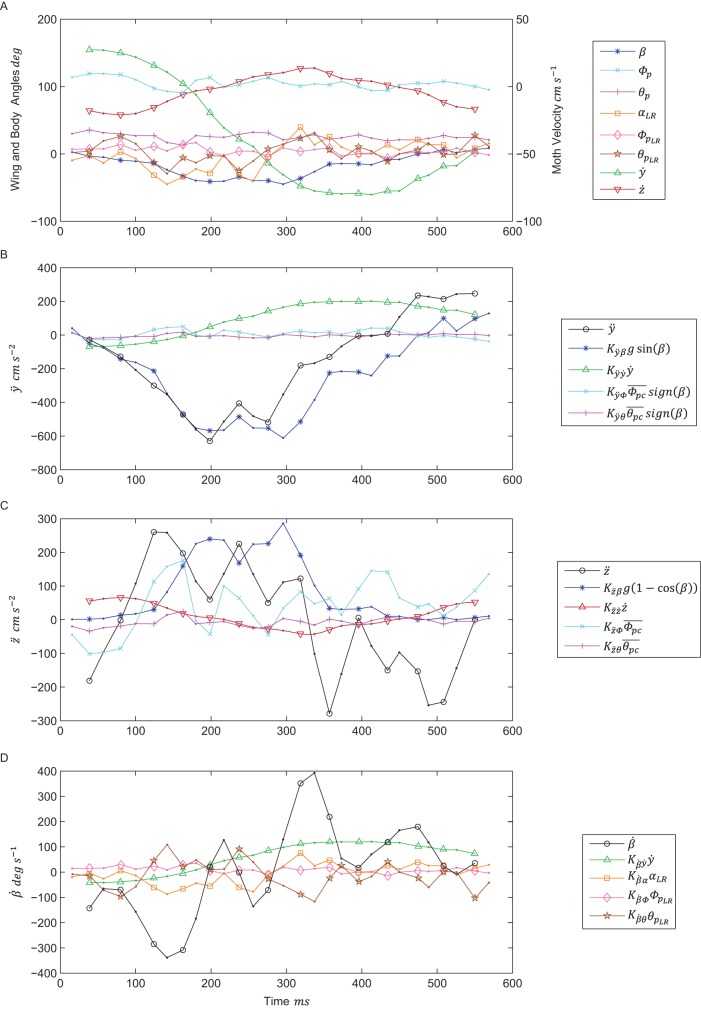
**Example trial segment.** (A) ≈580 ms (14.5 wingbeat) segment of midstroke body and wing kinematic data from a representative trial segment. This segment begins with a lateral deceleration followed by an acceleration in the opposite direction. (B-D) Estimated contribution of moth wing and body kinematics to *ÿ*, *z̈*, and 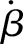, respectively. *ÿ*, 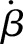, and *z̈* are in bold black, plotted against the independent variables from Eqns 1-3 after they are first multiplied by the estimated coefficients from the best linear mixed model of each orientation derivative. These coefficients are the same as those in Tables 1-2, and estimated from the entire data set. The plots show data at only midstrokes. Upstrokes are denoted by plain dots, while downstrokes are denoted by a different character for each measured variable.

Mixed models for 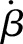 (roll velocity) show wing asymmetries affect roll velocity; primarily wing pitch angle (α), but also asymmetries in *Φ_p_* and *θ_p_*. Fig. 1 shows the results for *ÿ*, *z̈*, and 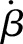, and highlights select relevant factors for these position and orientation derivatives. Fig. 2 shows kinematics for an example trial segment.

### AICc analysis results

Most predicted kinematics turned out to be significant. We subjected the mathematical models built to test our hypotheses to a variable selection process, as explained in Materials and Methods. Stepwise regression and AICc analysis for Eqns 6-8 (lateral acceleration, vertical acceleration, and roll velocity) reveals the highest quality models include the following variables (Eqns 1-3):
(1)
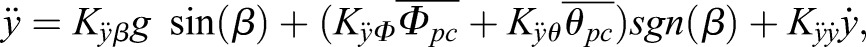

(2)
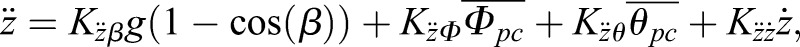

**Fig. 3.**
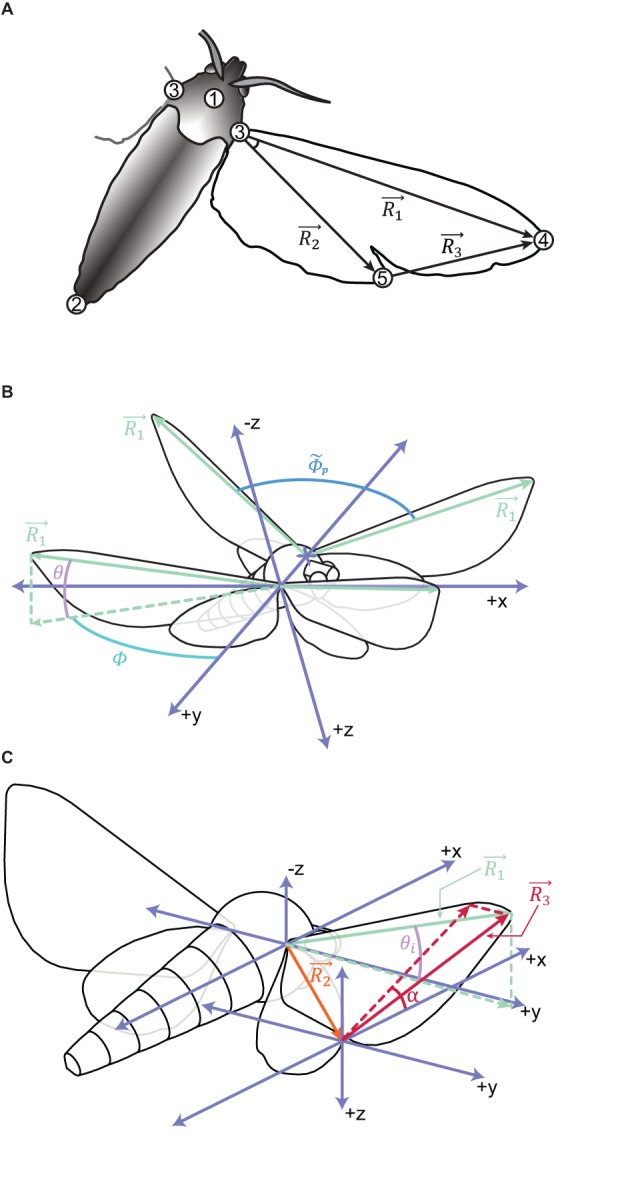
**Digitization and wing angle calculation scheme.** (A) Digitized points and resulting vectors used in wing position and orientation calculations. Only the body and right wing are shown in this image, but we marked points with bilateral symmetry on both sides of the sagittal plane. Point 1 was marked in an anterior portion of the scutum, point 2 at the tip of the abdomen, and point(s) 3 at the wing bases(s). We used points 1-3, measured in the GRF, to calculate yaw, pitch, and roll. These angles were then used to compute body point positions in the moth's BRF (Stengel, 2004). We next constructed an MGRF in which *z* remained vertical, but the *x*/*y* plane was rotated in yaw so that *x* was parallel with a vector running from the distal tip of the abdomen (point 2) to the geometric centroid of points 1-3, projected onto the GRF horizontal. In the MGRF, positive *x* movement is forwards for the moth, positive *z* movement is parallel with gravity (downwards), and positive *y* movement is to the moth's right. 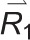 is the vector which stretches from the wing base point (3) to the forewing tip (4). 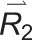 is the vector which stretches from the wing base point (3) to the hindwing tip (5), and 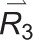 stretches from (5) to (4). (B,C) To compute wing pitch angle (*α*), we projected 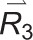 onto the BRF *x*/*z* plane and took *α* as the angle between this projected vector and the BRF horizontal. Midstroke wing pitch angles are all positive; we measured downstroke *α* relative to the positive moth BRF *x*-axis and upstroke *α* relative to the negative moth BRF *x*-axis. *θ* is the angle 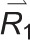 makes with the BRF horizontal; at midstroke, when measured ipsilateral to the direction of roll, we call it *θ_i_*. Peak-to-peak amplitude 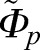 is the angle between 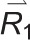
's BRF position at the top of upstroke and the end of downstroke, and *vv.* for the following halfstroke. Sweep amplitude, *Φ_p_*, is the projection of 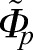 onto the BRF *x*/*y* plane, while *θ_p_* (not shown) is the projection of 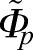 onto the BRF *x*/*z* plane.

(3)
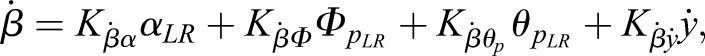
where each *K* is a linear coefficient relating the second subscript (the independent variable) to the first (dependent variable), *g*=980.665 cm s^−2^, and *sgn*(*β*) is ±1 according to the sign of roll orientation. Fig. 3 shows how we define our reference frames and angles. In the above equations, wing pitch angle is *α*, elevation amplitude is *θ_p_*, and sweep amplitude is *Φ_p_*. The symbols used to represent left+right wing pair means, vectors, first derivatives, second derivatives, peak-to-peak amplitude, the wing ipsilateral to moth lateral velocity, mean-centered data (mean of entire data set subtracted), and the left minus right orientation or amplitude difference are defined in Box 2.

**Box 1. List of abbreviations**

AICc: Corrected Akaike Information Criterion – evaluates model predictive quality while penalizing for model complexity
BRF: Body Reference Frame, where coordinates have been rotated to align with the moth's body axis such that forward movement by the moth is +*x*, rightward movement by the moth is +*y*, and upward movement by the moth is −*z*
DLT: Direct Linear Transformation: A method for extrapolating positions in space from pixels marked on captured frames from non-colinear camera views
FCT: Flapping Counter-Torque
GRF: Global Reference Frame: unchanged coordinates from direct linear transformation and alignment with handheld global axes, where −*z* is antiparallel with gravity
MGRF: Modified Global Reference Frame: reference frame which has been adjusted by rotating the GRF *x*/*y* plane so that the *x*-axis aligns with the yaw orientation of the moth
RML: Restricted Maximum Likelihood
RMSE: Root Mean Squared Error; in this study all RMSE values came from (data-relative) residuals.

**Box 2. General symbols**

 ¯: variable underneath is an average for the left and right wing combined
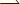
variable underneath is a vector
 ˙, ¨ : first and second time derivatives of the variable underneath, respectively
_*p*_: peak-to-peak amplitude of the antecedent variable
_*LR*_: differences in a kinematic measurement between the left and right side of a moth; i.e. left minus right
_*c*_: variable has been centered by subtracting its mean value for the entire data set
_*i*_: the wing ipsilateral to the direction of moth lateral velocity
*K*: any coefficient estimated by regression or mixed model
*g*: gravity, taken as 980.665 cm s^−2^
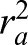
adjusted *r*^2^; calculated for linear models
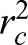
conditional *r*^2^; calculated for linear mixed models

**Box 3. Moth body kinematics**

*x*, *y*, *z*: front/back, lateral, and vertical (respectively) in the given reference frame
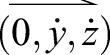
3D vector with moth horizontal and vertical velocity as its only nonzero components
*β*: moth whole-body roll angle, measured absolute to the GRF *x*/*y* plane

**Box 4. Moth wing kinematics**

See Fig. 3 for a detailed description of wing kinematics

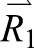
vector which stretches from the wing base point (point 3) to the forewing tip (point 4)
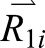
the 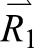 vector which is ipsilateral to the direction of moth lateral velocity
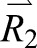
vector which stretches from the wing base point (point 3) to the hindwing tip (point 5)
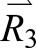
vector which stretches from the hindwing tip (point 5) to the forewing tip (point 4)
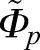
peak-to-peak angular amplitude
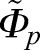
mean peak-to-peak angular stroke amplitude for left and right wings
*Φ*, *Φ*_*p*_: sweep angle; the projection of 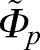 onto the BRF *x*/*y* plane
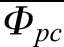
sweep amplitude averaged for left and right wings, then centered to overall data mean
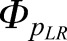
difference in sweep amplitude between the left and right wings
*Φ*_*LR*_: instantaneous difference in midstroke sweep angular position
*θ*: elevation angle; the angle 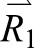 makes with the BRF horizontal
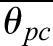
elevation angle, averaged for left and right wings, then centered to overall data mean
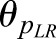
difference in elevation amplitude between left and right wings
*θ*_*LR*_: instantaneous difference in midstroke elevation angular position between the left and right wings
*θ*_*i*_: instantaneous midstroke elevation angle for the wing ipsilateral to moth lateral velocity
*α*: wing pitch angle; angle a projection of 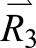 onto the BRF *x*/*z* plane makes relative to the BRF *x*/*y* plane
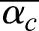
*α* averaged for the left and right wings, then centered to overall data mean
*α*_*LR*_: instantaneous difference in *α* between the left and right wings

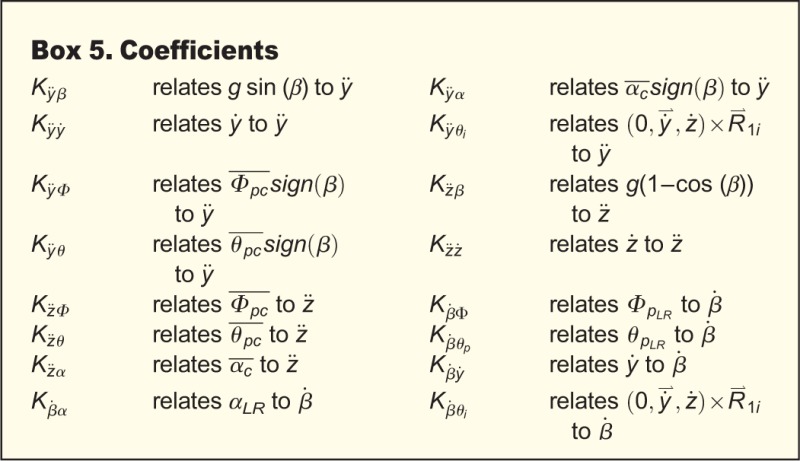


In our data, the AICc variable selection process does not eliminate any predictor variables from Eqns 6-8. The addition of a random intercept for each moth does decrease the minimum AICc value for both lateral and vertical acceleration models, and also brings *K_z̈β_* closer to its expected value of one. With the exception of 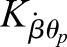, the coefficient relating elevation amplitude to roll velocity, all signs and magnitudes matched *a priori* expectations.

### Lateral and vertical accelerations

Basic gravitational predictions are highly significant; wing asymmetry predictions are also significant, but less so. In order of significance, the lowest AICc lateral acceleration model (Eqn 1) includes sin(*β*), *ẏ*, 
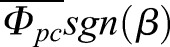
, and 
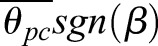
, while the lowest AICc vertical acceleration model (Eqn 2) includes 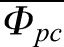, 1−cos(*β*), *θ_pc_*, and *ż*. The best fit models for *ÿ* and *z̈* are linear mixed models (i.e. they contain corrections for individual moth effects), while the best AICc model for 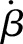 is a linear model. See Table 1 for the coefficients estimated for the best fit models as well as coefficients and *P*-values for full variable linear models *prior* to variable selection. For all of these independent variables, coefficient signs and magnitudes match expectations as outlined in Materials and Methods. We also calculated angular stroke amplitude (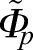) as the relative angle between the wingtip at the end of each halfstroke in the BRF. We compared the AICc and *P*-values of Eqns 6-7 against modified versions where we replaced 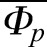 and/or 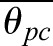
with 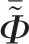. The results of these comparisons show better AICc values for 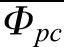 alone, as well as 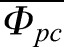 and 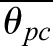 together as a unit, than equations with 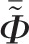. The significance of *K_ÿΦ_* and *K_ÿθ_* is not a result of their *sgn*(*β*) multiplier; if we attempt to fit 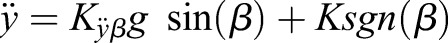, the *P*-value for *K* is not significant. Mixed models improve the most when we subtract the mean 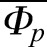 and 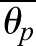 for the entire data set, rather than for each individual moth or trial. See Table 2 for a comparison of the contribution of each of these effects relative to one another.

**Table 1.**
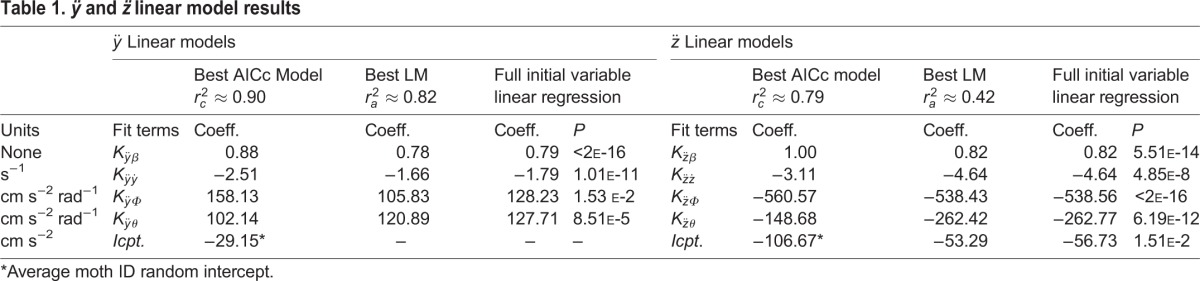
***ÿ* and *z̈* linear model results**

**Table 2.**
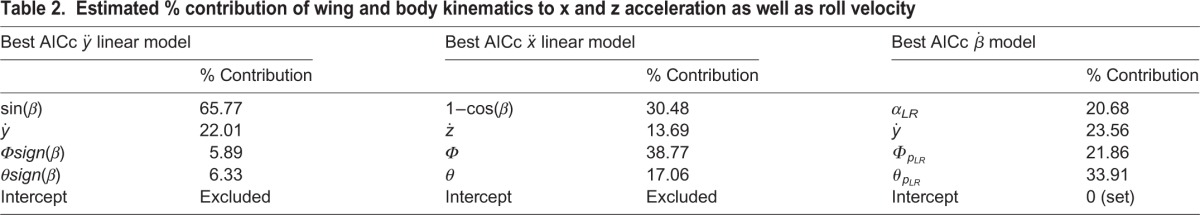
**Estimated % contribution of wing and body kinematics to *x* and *z* acceleration as well as roll velocity**

### Roll velocity

For reasons detailed in Materials and Methods, we present model results for roll velocity rather than roll acceleration. The best AICc model for roll velocity (Eqn 3) includes *α_LR_*, 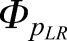, 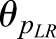, and *ẏ*, the signs of 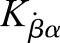, 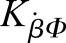, and 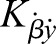 match *a priori* expectations (see Materials and Methods), but the sign of 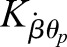 does not. See Table 3 for these coefficients and Table 2 for a comparison of the contribution of each of these effects relative to one another. Instantaneous left-right wing position differences at midstroke separately correlate with roll velocity; however, such wing position differences do not appear in the best AICc models – likely because they simply recapitulate 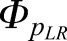, 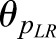, and *α_LR_* (possibly with additional noise).

**Table 3.**
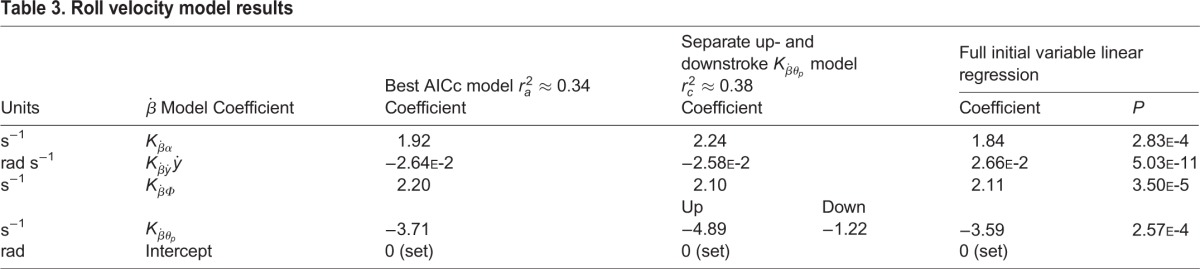
**Roll velocity model results**

Moths show a stair-step pattern in roll at endstrokes; they reorient more in the overall direction of roll during downstroke and less during upstroke (Fig. S1). In fact, roll in the overall direction of motion is often lost in upstroke rather than gained. This stair-step pattern corresponds to periodicity in *α_LR_* (Figs S2, S3). Autocorrelations show *α_LR_* typically holds the same sign for consecutive wingbeats but displays periodicity in magnitude (Fig. S3). Average downstroke 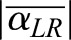 is 1.57 times upstroke 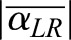, and downstroke α is 1.30 times upstroke α. Conversely, *Φ_p_*, 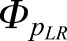, *θ_p_*, and 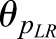 are about the same magnitude and hold consistent sign for consecutive half and whole wingbeats.

### Computational model results

Blade element model (Hedrick and Daniel, 2006) results mostly match observed trends. Velocity decay half-lives (i.e. time constants), estimated from the differential solutions to Eqns 1-2, are similar to those extracted from two computational models of passive theoretical *M. sexta* (Hedrick and Daniel, 2006; Kim and Han, 2014). Coefficients values from observed data and the blade element model also agree (Tables S2, S3). Unfortunately, the model is not sufficiently accurate to test whether the wing kinematic changes we observed fully create the body movements we observed. However, it was useful in interpreting whether the approximate wing kinematic changes selected in the experiments create the same general movements in the model as they seem to do in actual moths.

## DISCUSSION

### Summary

*M. sexta* can sideslip, and they do so by rolling their body to reorient their net force vector. They augment the net force they produce during sideslips to prevent sinking by increasing flapping amplitude, and encounter decelerative drag proportional to their lateral and vertical velocity. Roll maneuvers are multifactorial and involve a high degree of damping.

These moths create roll torque via left-right asymmetries in sweep amplitude, elevation amplitude, and most importantly, midstroke wing pitch angle. Because moths are heavily roll-damped, asymmetries in flapping kinematics at the half-wingbeat timescale relate linearly to the first, rather than second, derivative of roll (when roll is measured at that same timescale). In addition to flapping counter-torque (FCT), this damping torque likely at least partially originates from induced angle of attack asymmetries – a well-known effect in rolling fixed-wing aircraft. Moths roll more in the direction of net reorientation during downstroke than upstroke by modulating the wing pitch angle difference in each halfstroke, and potentially due to larger upstroke FCT since wing pitch angles and their left-right differences are smaller in upstroke.

### Lateral and vertical acceleration

Our models of lateral and vertical acceleration indicate *M. sexta* roll to redirect their net force vector and thus create lateral acceleration. The most significant relationship between independent and dependent variables in the entire study is that between lateral acceleration and the moth's whole-body roll angle [*g* sin(*β*) and *ÿ*]. The relationship between roll orientation and vertical acceleration [*g*(1−cos(*β*)) and *z̈*] is also highly significant. The coefficient for this latter relationship (*K_z̈β_*) is positive because −*z* in our coordinate system is antiparallel with gravity (upwards). The magnitudes of *K_ÿβ_* and *K_z̈β_* are both very close to one, as expected. These relationships were also predicted by *a priori* hypotheses and further supported by visual inspection. The strong match between the roll-based sideslip hypothesis and the lateral acceleration data indicates that any additional effects such as direct lateral force production via left-right asymmetries in flap timing produce only marginal forces if they are present at all. Our linear mixed model based on roll only accounts for roughly 90% (based on 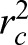) of the observed lateral acceleration. We did consider that our use of a moving light source positioned above the moth may have enhanced the roll response in these recordings compared to self-motivated sideslips, cueing the moths to rotate their body and head to maintain a constant visual angle to the light source. However, we consider this unlikely since head stabilization is evident in our videos, and from a digital comparison of antennae position relative body orientation.

On the time scale of half wingstrokes and over the airspeed range we observed, *M. sexta* is a physical system in which we can model damping opposite the direction of lateral and vertical motion as approximately proportional to collinear velocity. Comparisons between coefficients and time constants estimated from mathematical models and observed data (Tables S2, S3) show that this resistance probably comes from passive drag, though some resistance could conceivably come from active steering by the moth in an attempt to limit acceleration.

Finally, our linear acceleration results show that moths flap with greater amplitude to increase net maneuver force and maintain altitude during sideslips. This is opposed to alternative possibilities of bilaterally increased wing pitch angle or flapping frequency; neither of which significantly relate to increased acceleration. Increased wing pitch angle may present a problem for hawkmoths, since they already use high effective angles of attack while hovering, (Ellington, 1984a,b; Ellington et al., 1996), and even higher angles when they use wing pitch asymmetry to create roll torque (Table S4). Moths have shown small (∼2 Hz) increases to flapping frequency in response to wing clipping (Fernandez et al., 2012), though in the absence of wing area alteration, their flapping frequency (∼26 Hz) may be close to some physiological limit; unclipped moths show insignificant increases in flapping frequency when maneuvering at speed (Ortega-Jimenez et al., 2013).

### Roll velocity

The biggest active contributor to roll initiation is wing pitch angle, a relationship estimated by coefficient 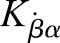 (Eqn 3). The positive signs of 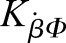 and 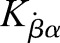 show that greater wing pitch angle and sweep amplitude on one side of the moth relative to the other creates roll torque away from that wing pair (contralateral roll). Since airfoil velocity and angle of attack affect lift force, this follows well with established theory. Based on AICc results comparing models which used wing pitch angle calculated in several different ways, we believe the way we measure *α* (Fig. 3) is the best way to represent the kinematic relationship with the data we have. One discarded alternate wing pitch angle measurement was based on a wing ‘chord’ stretching from the hindwing tip to a perpendicular intersection with 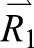 (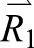 is shown in Fig. 3). Precise estimates of actual effective angle of attack for *M. sexta* could have been better; unfortunately such estimates suffer from a number of confounding factors, such as increased noise, variations in wing curvature, and the complexity of *M. sexta* wing air flow dynamics, (Ellington et al., 1996; Bomphrey et al., 2005; Zheng et al., 2009).

We delved deeper into single-wing kinematics to determine precisely how hawkmoths manifest the wing asymmetries which create roll. To manifest the wing pitch asymmetry, a moth rolling to the right alters left and right wing pair *α* by about the same amount but in opposite directions; increasing the left while decreasing the right (Table S4). Instantaneous midstroke asymmetries in sweep and elevation angular position (*Φ_LR_* and *θ_LR_*) also correlate strongly and positively with roll velocity; as expected if amplitude asymmetries are unevenly distributed about the midline. A positive correlation between instantaneous midstroke sweep angle and roll velocity suggests that, to manifest sweep amplitude asymmetries, a moth rolling to the right decreases 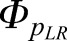 primarily by extending its right wing pair relatively less far forward in comparison to its left wing pair. Meanwhile, the positive correlation between instantaneous midstroke elevation angle and roll velocity is more ambiguous, since the measurement of midstroke *θ_LR_* is linked to changes in *α_LR_*. Binned average values (Table S4) imply it is possible moths bilaterally adjust both sweep and elevation angle amplitude much as they do wing pitch angle, but *t*-test results for this are not significant.

We observe oscillation in both 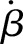 (in the form of a stair-step pattern) and *α_LR_* for up- vs downstrokes (Fig. S3). Thus, we here report results for mixed models which separate up- and downstroke coefficient estimations. All derivatives depend on stroke-to-stroke changes rather than instantaneous measures so this halfstroke-frequency stair-step pattern did not interfere with how we calculated coefficients. Models where we introduced separate 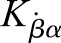

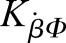
, and/or 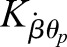 for up- and downstrokes are not better according to AICc. Note *α_LR_* is higher for downstrokes but 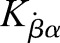 remains the same. This strongly suggests moths create more roll torque in the target direction of movement in downstrokes rather than upstrokes, at least in part, by adjusting the magnitude of *α_LR_*. A further plausible explanation for the stair-step pattern in roll comes from key kinematic differences between up- and downstroke which likely result in greater upstroke damping, as discussed next.

### Roll damping and FCT

Results here compliment wing velocity mediated roll damping described in the turning free-flight of cockatoos, (Hedrick et al., 2007), and computational studies which predict heavily damped roll in *M. sexta* (Kim and Han, 2014). Effective angle of attack asymmetry induced by rolls, as in fixed-wing aircraft, almost assuredly dampens movement in moths as well. Our results further suggest FCT effects. The velocity of the wings about the roll axis is additive with velocity created by overall body and wing reorientation in the global reference frame (GRF). This decreases lift in the wing contralateral to a roll, and increases lift on the wing ipsilateral to a roll (Hedrick and Biewener, 2007; Hedrick et al., 2009). Flapping counter-torque is a drag effect, where cross-sectional area of the wing relative to the rotation, in part, determines the strength of the effect. Thus, *cet. par.*, FCT (and thus roll damping) depends on the inverse of wing pitch angle as defined in this work. The data show wing pitch angle magnitudes and asymmetries are both smaller in upstrokes than downstrokes (Figs S2, S3). So, we would predict increases in elevation amplitude have a bigger negative impact on roll during upstrokes. Consistent with this roll FCT explanation, linear models in which we separate up/downstroke *θ_p_* result in a more negative upstroke 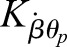 (Table 3). Our results here agree with an FCT explanation. They contradict our *a priori* expectation that increased roll-contralateral *θ_p_* (relative to ipsilateral) would increase relative contralateral force and thus 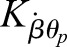 would be consistently positive, and presumably larger for downstrokes.

We find evidence of antagonistic coupling between roll and lateral velocity, (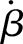 and *ẏ*), where roll velocity towards a given side negatively correlates with whole-body velocity in that direction (i.e. rightwards roll velocity correlates negatively with rightwards linear velocity and *vv*). As seen in Fig. 2, our data include both lateral accelerations and decelerations, and the negative correlation between lateral velocity and roll velocity is significant for both cases. In lateral accelerations (sideslip initiation) the ipsilateral wing pair moves towards shed air, while the wing pair contralateral to sideslip moves away, which may increase ipsilateral wing force. In lateral decelerations, (sideslip reversal), lateral velocity may negatively correlate with roll velocity simply since moths are rolling away from their direction of sideslip in order to redirect force and slow down. As an alternative or additional explanation, this antagonistic coupling in lateral acceleration specifically is also suggestive of the velocity-mediated sideslip damping proposed by Faruque and Humbert (2010).

### Comparison to aerial maneuvers in other animals

Given previous research, we can compare the sideslip maneuvers of *M. sexta* directly to sideslips of fruit fly *D. melanogaster* and mosquito *A. aegypti*, and indirectly to other maneuvers in birds and bats. Firstly, our results do not support a scenario in which *M. sexta* wing pitch angle timing asymmetries at the ends of halfstrokes play a prominent role in creating direct lateral force, as in *D. melanogaster* (Ristroph et al., 2009). Preliminary examination of videos digitized continuously through the wingbeat cycle does not show evidence of consistent timing differences. Instead our data provides strong support for the simple body roll hypothesis. Thus we conclude moths use roll to reorient their net force vector, much like birds performing turns (Hedrick and Biewener, 2007; Hedrick et al., 2007; Ros et al., 2011), and mosquitoes performing sideslips (S. Iams, PhD thesis, Cornell University, 2012). Iams measures ‘stroke plane roll’, the angle a line between the two wingtips makes relative to the horizontal. In our moths, a Pearson product moment correlation of body roll with stroke plane roll measured at midstroke yields 0.944, indicating high agreement between the two measures. This suggests stroke plane roll is indeed a plausible measurement to use in place of body roll to determine the direction of net force creation in the *y*/*z* plane. *D. melanogaster* also uses a tilted stroke plane roll angle to create a portion of its lateral acceleration during sideslips and saccades (Ristroph et al., 2009; Muijres et al., 2015).

Free-flight *M. sexta* rolls are also comparable to aerial rolls in other animals. A multitude of land animals use their tail and body to perform inertial reorientation to right themselves in the air (Jusufi et al., 2011). Despite *M. sexta* possessing a weighty ‘tail’ in the form of a flexible abdomen, we did not observe it to have a role in roll reorientation. This contrasts with abdominal reflexes recorded in *M. sexta* in response to pitch or yaw displacement (Dickerson et al., 2014; Dyhr et al., 2013; Hinterwirth and Daniel, 2010), modeled effects on stability (Noda et al., 2013), as well as the active aerodynamic role of the flat tails of birds and bats (Adams et al., 2012; Gardiner et al., 2011; Thomas, 1993). Instead, *M. sexta* appears to rely solely on the aerodynamics of its flapping wings to create roll maneuvers – and not at all on its wing inertia, which contrasts with both turning pigeons (Ros et al., 2011) and reorienting bats (Bergou et al., 2011; Iriarte-Diaz et al., 2011). To create roll torque, moths use a combination of wing stroke asymmetry, as seen in turning birds (Hedrick and Biewener, 2007; Ros et al., 2011), and wing pitch asymmetry – similar to wing camber/pitch asymmetry observed during downstroke in both the aforementioned birds as well as dragonflies and hummingbirds (Altshuler, 2012; Wang et al., 2003); but dissimilar to the wing rotation angle asymmetries which create roll torque in fruit fly saccades (Muijres et al., 2015). Like our moths, hummingbird *Calypte anna* has been shown to roll while turning to influence lateral velocity, and that they: (1) increase the mean elevation angle; (2) decrease the elevation amplitude; and (3) increase the stroke amplitude of the contralateral wing relative to the ipsilateral wing (T. J. G. Read, MSc thesis, University of British Columbia, 2015, https://open.library.ubc.ca/media/download/pdf/24/1.0166172/1). However, unlike our moths, they showed a timing difference in wing pitch angle between the contralateral and ipsilateral wings which did not manifest as an angle difference at mid-downstroke.

The stepwise nature of roll reorientations and the significant relationship of moth wing kinematic changes with roll velocity rather than acceleration, together suggest a highly damped, roughly first order system for roll; which agrees with prior free-flight research on both roll and yaw in flapping flight. Previous research on hawkmoth yaw turns revealed a roughly first-order control system in which imbalanced force from stroke amplitude and wing angle of attack asymmetry drives yaw rotations, and imbalanced drag on the wings induced by the yaw rotation naturally dampens the physical system (Hedrick et al., 2009; Ortega-Jimenez et al., 2014). Since the component of *M. sexta*'s wing velocity about the roll axis (which creates FCT in roll) is much smaller than the component about the yaw axis (which creates FCT in yaw), the existence of a first-order relationship between wing kinematics and body roll is especially interesting. The apparent existence of a first order system in *M. sexta* in the mediation of roll, supported by our live-animal data as well as computational models, in addition to yaw, suggests that first order control relationships could be the norm for body orientation control in flapping flight.

## MATERIALS AND METHODS

### Animals

We acquired four male *M. sexta* as pupae from a domestic colony at the Duke University Department of Biology. These *M. sexta* were from a line of hawkmoths which was recently out-crossed with domesticated lines from several other universities. Following eclosure, adult moths had access to honey dissolved in water *ad lib.* They lived in (30×30×30 cm) cubic mesh cages and were kept on an extended day, abbreviated night light cycle. Moths were between one and fifteen days *post*-eclosure at time of use. See Table S5 for individual moth morphological details.

### Experimental setup

**Fig. 4.**
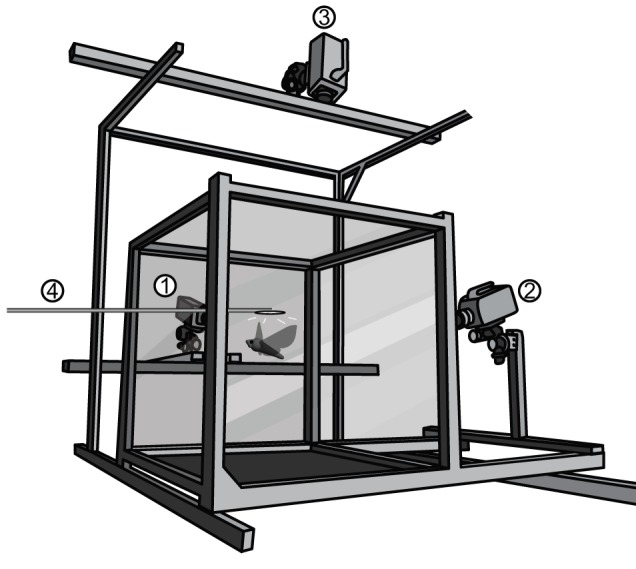
**Flight chamber and cameras used in these experiments.** (1, 2) Phantom v7.1 cameras; (3) Phantom v5.1 camera; (4) oscillating light source.

We recorded moth sideslip maneuvers from hawkmoths flying in a 71×71×74 cm glass-walled arena following an oscillating light (Fig. 4). Two Phantom v7.1 and one Phantom v5.1 digital cameras (Vision Research Inc., Wayne, NJ, USA) used the high-intensity 680 nm light from eight LEDs (Roithner LaserTechnik, GmBH, A-1040, Vienna, Austria) to capture moth maneuvers at a frame rate of 600-700 Hz. We filmed trials at night inside a closed, unlit room with shuttered windows. The ambient light level in the filming room was approximately 180 lx at the time of filming, and only 10 lx without the high-intensity infrared LEDs, as measured with a lux meter (840006, Sper Scientific LTD, Scottsdale, AZ, USA). The time of filming generally coincided with nighttime in the moths' abbreviated night/day cycle. Most moths warmed up and flew naturally in the dark flight chamber, but some required manual stimulation with thumb and forefinger to elicit warm-up behavior. Once the moths were hovering at least one wingspan above the floor of the chamber, the light was oscillated above the moths, horizontal to the ground and roughly perpendicular to the moths' sagittal plane with an approximate frequency of 1.25 Hz and peak-to-peak amplitude of 25 cm. To construct the light, we mounted a 2.2 cm radius cut-out of a phosphore/dialectric light (model# 11100, 115 V/0.03 W, EI Products Inc., Maxwell, TX, USA) on the end of a 50.7 cm long metal rod. The moths exhibited phototaxis and spontaneously followed the light's path (see Movie 1).

### Camera calibration

We used direct linear transformation (DLT) to calibrate the cameras (Hedrick, 2008). The DLT input points were the filmed pixel positions of two light-emitting diodes situated 68.5 mm apart at the end of a wand, after we waved this wand through the filming space by hand. Three calibrations were used among the different recordings. Their pixel (*u, v, w*) Calibration Root Mean Squared Errors (RMSE) for each of the three cameras were (0.12, 0.14, 0.14) for trials 1-2, (0.15, 0.11, 0.10) for trials 3-6, and (0.13, 0.14, 0.15) for trial 7. See Table S5 for the median RMSE of each digitized point for each trial. We based the first calibration on wand points we tracked by hand, and the second two calibrations on wand points tracked by custom software.

### Video data analysis

We used qualitative observations of raw video data to select seven trials in which the moths were sufficiently visible for manual digitization and underwent minimal yaw rotation throughout their individual sideslip maneuvers. These videos are comprised of 19 distinct lateral maneuvers; defined as lateral accelerations where the direction of acceleration is sustained for at least 0.077 s, or about two 26 Hz wingbeat cycles, which is approximately the average wingbeat frequency in our trials.

### Measuring wing parameters

We marked points at each of four visually-identified phases in the moths' wingstrokes: (1) end-downstroke, (2) end-upstroke, (3) mid-downstroke, and (4) mid-upstroke. This allowed us to identify key points in the stroke cycle for analysis and decreased the requisite amount of manual digitizing. In each of these four frames, we digitized 8 moth body and wing points (Fig. 3) using the MATLAB (r2011a, The Mathworks, Natick, MA) package DLTdv5 (Hedrick, 2008). We (rarely) excluded point 5 when visibility did not allow us to digitize it. Both the left and right wing points were marked independently on every digitized frame.

We used MATLAB to compute Euler angles and wing kinematics from the digitized points. Fig. 3 shows wing angles and explains reference frames, including the modified global reference frame (MGRF), GRF, and BRF. To filter out regular within-wingbeat fluctuations, we calculated position and orientation derivatives from wingbeat to wingbeat changes only; we measured changes between like points in the flapping cycle rather than from each digitized frame to the next. We then averaged the resulting four derivative measurements (since there were four digitized frames per stroke) to capture both high and low frequency changes not tied to within-wingbeat oscillations. We fit *x*, *y*, *z*, and their derivatives, (i.e. whole-body position and movement), in the MGRF only. When correlating these body movements with wing kinematics, we inserted stroke amplitude values at their corresponding midstroke points. This is where we focused analysis since midstrokes are a convenient reference point where forces are either highest, or close to their highest, in the *M. sexta* stroke cycle (Bomphrey et al., 2005; Zheng et al., 2013). We calculated all angles using points from the BRF.

### *A priori* lateral and vertical acceleration models

Here we describe the simplified models we used to analyze links between three aspects of moth movement, including lateral and vertical acceleration, as well as roll velocity. We started with the following two equations, which are based on a first principles, constant dorsally-directed force (equal to body weight) model of animal flight as follows:
(4)
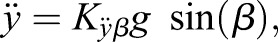

(5)
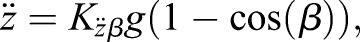
where *β* is measured relative to the MGRF *x*/*y* plane. Eqn 4 tested our hypothesis that moths roll to accelerate laterally, while Eqn 5 tested the conjugate force model for vertical acceleration. For all equations *y*, *z* and their derivatives are in the MGRF, i.e. aligned to moth sideslip motion and gravity, respectively. At no point did we attempt to separate the initiation of acceleration and its reversal; we fit the data with the same linear models regardless of the direction in which the moths were attempting to accelerate/decelerate.

### Linearizing resistive forces and adding other model terms

We added to these basic equations kinematic variables which we had *a priori* reason to expect might contribute to moth directional movement, and then applied a stepwise variable elimination approach. Here, 
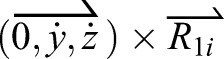
, which is roughly equivalent to 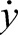 sin (*θ_i_*), represents drag on the wing ipsilateral to the directional or rotational movement. We attempted to fit this kinematic because the wing ipsilateral to the movement direction is exposed to both lateral velocity and roll velocity. We report the attempted fit of this measurement for completeness since we used all attempted kinematics when calculating *P*-values. Since we are interested in how departures from typical flapping leads to the creation of movement, we used the mean-centered versions of 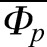 and 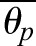. In Eqn 6, subtracting the overall mean isolates variance and thus allows us to multiply by *sign*(*β*) to estimate coefficients. In Eqns 7-8, mean-centering 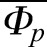 and 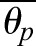 allows us to assume a zero intercept; a significant intercept result would indicate moth-specific variation or that our model fails to represent the complete moth system maneuver dynamics.

In the most general sense, high Reynolds number air drag is proportional to velocity squared. However, the velocities moths encountered in our experiments cover a small range, over which we might expect to reasonably linearize an exponential trend. Furthermore, computational analysis of the flight of *M. sexta* suggests that, on the time scale of half wing-strokes, passive resistance to movement during horizontal and vertical movement is roughly linearly proportional to velocity rather than velocity squared for both horizontal and vertical movement terms, (Cheng and Deng, 2011; Kim and Han, 2014). Further velocity damping effects have also been proposed (Faruque and Humbert, 2010). Linearizing the resistive forces and adding other model terms resulted in the following equations, (see Expected coefficient values for predictions):
(6)
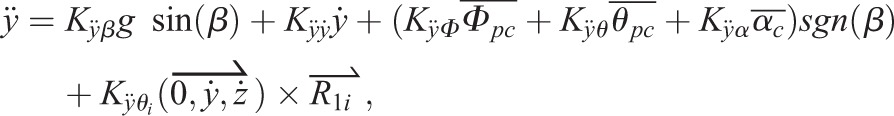

(7)
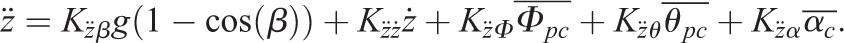


### Fitting roll dynamics

It quickly became clear the data strongly supported Eqns 4,5, so we next investigated *β* (roll). Final models involve the first, rather than second, derivative of roll. Here we justify this choice.

A combination of factors led us to fit the first, rather than the second derivative of roll. Both previous and concurrent works predict strong damping in roll during flapping flight (Hedrick, 2011; Kim and Han, 2014). Trials 1-2 were continuously digitized and preliminarily analyzed; as expected, the results show accelerations which vary greatly throughout each halfstroke, and even more over the course of whole wingstrokes. Yet wing asymmetries which correlate with roll are largely conserved from each halfstroke to the next, and roll velocity direction is largely conserved from each whole stroke to the next (Figs S1-S4). This indicates sustained intended direction of reorientation, and allows us to ignore accelerative changes on the sub-halfstroke time scale. Not only did prior kinematic analysis of *M. sexta* performing yaw turns also fit the first rather than second orientation derivative (Hedrick and Robinson, 2010), but recent computational models indicate that roll on the scale of half-wingstrokes experiences heavy damping such that a linear roll-velocity model may actually be most appropriate (Cheng and Deng, 2011; Kim and Han, 2014). Regardless, we did attempt to fit roll acceleration for completeness. Linear regressions of the second derivative of roll versus various wing asymmetries reveal no significant trends.

We did not have a first principles prediction for the kinematics behind roll velocity. So, we compiled a preliminary model which related various wing angle differences and body kinematics we suspected may be important to roll, and then applied the same stepwise variable elimination approach. We verified the wing angle correlations visually in several instances before adding them to the model.
(8)
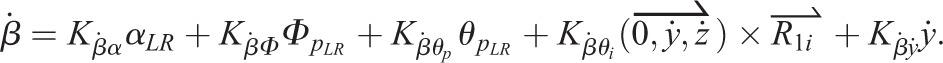


### Expected coefficient values

In Eqns 6-7, we expected the values for both *K_ÿβ_* and *K_z̈β_* to be close to +1. We expected *K_ÿÿ_*, *K_z̈ż_*, and 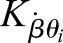 to be negative; where the component of velocity antiparallel to acceleration – or, in the cases of 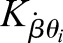 and 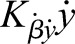, ipsilateral to the moth's direction of rotation – dampens motion. Since greater flapping amplitude increases force, we expected *K_ÿΦ_* to be positive and *K_z̈Φ_* to be negative (positive *z* is down). To account for the expected nature of *Φ_pc_*'s contribution to *ÿ*, we multiplied *K_ÿΦ_* by the sign of *β* before fitting *K_ÿΦ_*. We expected 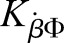, 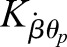, and 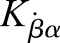 to all be positive since we expected more vigorous flapping and higher angle of attack on the left side of the moth should send the moth rolling to the right, and *vv*.

### Statistics

We performed initial regressions in MATLAB, and final mixed model analysis in R (R Development Core Team, 2013). After selecting variables in the kinematic equations using stepwise variable elimination, we used AICc in conjunction with linear and linear mixed models to identify those of the best quality. To assess model quality, AICc evaluates how closely a model's predictions match observed data while penalizing for complexity. As explained later in this section, we used a cascade approach, rather than a full variable sweep, to choose and compare models.

For all models, we retroactively attempted autoregressive correlation structures with *corAR1* and *corARMA* from the *nlme* library (https://cran.r-project.org/web/packages/nlme/nlme.pdf) to evaluate the necessity of adjustment for the time series nature of the data. We used the *AICc* function from the *AICmodavg* library (https://cran.r-project.org/web/packages/AICcmodavg/AICcmodavg.pdf) to evaluate the AICc values for each linear mixed model. For lateral and vertical acceleration models, all attempted autoregressive correlation structures resulted in erratic residual behavior and increased AICc, indicating reduced model quality. Since these basic autocorrelation structures did not improve fits, we did not evaluate more sophisticated techniques like vector autoregression nor apply any autoregressive correlation structures to the final chosen models.

Our cascade AICc comparison approach started with a series of linear models, using R's *lm* function from its *stats* library (R Development Core Team, 2013) with the *qr* optimizer from the *nlme* library (Pinheiro et al., 2014). We started by testing the most significant identified variable against the null hypothesis of a simple intercept. For Eqns 1,2, we also attempted to fit a variety of intercepts, since scatter was low, and because we qualitatively observed ample variation in typical sweep and elevation amplitude among moths. In the case of roll velocity (Eqn 3), we did not fit an intercept since one would expect no wing kinematic asymmetries in the case of a stably hovering moth, and no roll-damping affect for zero lateral velocity. Once the most significant variable was confirmed to decrease AICc, we added the next least significant variable to the model and tested whether it additionally improved AICc values, but also recursed (at least) one step by additionally testing the model with the lone exclusion of the variable confirmed in the previous step. In this way we proceeded until we had tested that all variables identified in the stepwise variable elimination improved model quality.

Once the linear model cascade was complete, we then attempted to fit linear mixed models using *lme* with the *optim* optimizer and *REML* estimation technique, (also from *nlme*), (Pinheiro et al., 2014). Here, we evaluated models of random intercepts for two possible factors in the analysis: moth and trial number in the case of lateral and vertical acceleration; and the same two intercepts as well as random coefficients for up- and downstroke in the case of roll velocity. This tested whether allowing for fixed variations between individual trials, moths, or (in the case of roll velocity) halfstrokes improved model quality. We attempted to fit random intercepts and coefficients for each possible combination of variables which had been shown to improve model quality. To limit complexity, we did not attempt nested group structures.

To correct for our initial variable selection with stepwise linear regression, we report *P*-values (Tables 1, 3) for linear models in which all initially attempted independent variables are used at once, (Eqns 6-8), regardless of their significance. We used *summary* from the *stats* library to calculate the *P*-values for each coefficient. However, when two initially tested measurements were extremely similar in nature, such as wing pitch angle and estimated effective angle of attack, we only represented it once in the linear model fit which determined its *P*-value. We report *P*-values for the initially attempted models, rather than report artificially low *P*-values in models composed only of independent variables for which we found significant correlations with the dependent variable. We extracted adjusted *r*^2^ (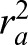) values directly from the output of the *lm* function, and evaluated the conditional *r*^2^ (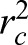) value of mixed models using the *r.squaredGLMM* function from the *MuMIn* library (Barton, 2015).

To understand more precisely how moths alter their wing dynamics to create roll, we also compared normal flapping to that during maneuvers. We treated observed roll velocities in the bottom 25th percentile as normal flapping, and designated the rest as high roll velocity flapping. Then we used MATLAB's *t*-test (paired *t*-test) to compare the movements of the ipsi- and contralateral wings during high roll velocities with wing movements during normal flapping, and *t*-test2 (unpaired *t*-test) to compare high roll velocity ipsi- and contralateral wing movements to one another (Table S6).

For both the roll velocity and linear acceleration models, we averaged the coefficient times the average absolute value of the kinematic measure and added them all together to create a reference value to determine percentage contribution as seen in Table 2.

### Blade element model

We used a blade element model to investigate the effects of measured wing kinematics on a computationally simulated hawkmoth (Hedrick and Daniel, 2006). This model sums quasi-steady estimates of aerodynamic forces due to rotation of the wing about its spanwise axis (Sane and Dickinson, 2002), wing translation (Dickinson et al., 1999), and added mass. We modified the simulation from Hedrick and Daniel (2006) to include independent kinematics for the left and right wing pairs. We used basic flapping parameters which match those of a hovering hawkmoth. We investigated the different kinematic adjustments observed in the moths by modulating either the amplitudes or, when differences were reported for mid-stroke only, by modulating the mean. We modulated the means and amplitudes since this produces wing kinematic changes without altering the relative phases of the left and right wing pairs. For example, we produced a 10 degree change in wing pitch angle at mid-stroke by increasing the average wing pitch angle of the right wing pair by 5 degrees and decreasing the wing pitch angle of the left wing pair by 5 degrees.

### Analysis of methods and limitations

Methods were sufficient to test our initial hypotheses, and led to secondary hypotheses about roll which we were also able to test; but, there were some shortcomings. The data size was insufficient to determine the simultaneous relevance of multiple nested random factors (i.e. maneuver, trial, moth number, and up- vs downstroke), since using *lme* with this group structure often resulted in overfit data in the form of singularities.

Several independent variables which we had little physical reason to believe should correlate to observed kinematic trends yielded significant *P*-values. Unlike expected independent variables which also had significant *P*-values, these unexpected independent variables increased minimum AICc values in the models. This further validated our approach of using the AICc cascade approach for checking expected relationships, even though it did not eliminate any variables which survived stepwise regression variable selection.

We were unable to separate the wing kinematics of the moth's compensatory control of yaw from its intentional creation of roll. This problem is especially confounding since previous studies and computational model data indicates the wing kinematics that we show effect roll should also effect yaw (Hedrick et al., 2009; Hedrick and Robinson, 2010; Ortega-Jimenez et al., 2014). Yet, understanding the specifics of yaw control is not a question we designed this experiment to answer; but rather one we plan to address in future research.
